# Influence of macrolide maintenance therapy and bacterial colonisation on exacerbation frequency and progression of COPD (COLUMBUS): Study protocol for a randomised controlled trial

**DOI:** 10.1186/1745-6215-13-82

**Published:** 2012-06-09

**Authors:** Sevim Uzun, Remco S Djamin, JanAJW Kluytmans, Nils E Van’t Veer, Anton A M Ermens, Aline J Pelle, Paul Mulder, Menno M van der Eerden, JoachimGJV Aerts

**Affiliations:** 1 Department of Respiratory Medicine, Amphia Ziekenhuis, Breda, The Netherlands; 2 Department of Microbiology, Amphia Ziekenhuis, Breda, The Netherlands; 3 Hospital Pharmacy, Amphia Ziekenhuis, Breda, The Netherlands; 4 Laboratory for Clinical Chemistry and Hematology, Amphia Ziekenhuis, Breda, The Netherlands; 5 Center of Research on Psychology in Somatic Diseases, University of Tilburg, Tilburg, The Netherlands; 6 Department of Biostatistics, Erasmus Medical Centre, Rotterdam, The Netherlands; 7 Department of Respiratory Medicine, Erasmus Medical Centre, Rotterdam, The Netherlands

**Keywords:** COPD, COPD exacerbation, Macrolide antibiotics, Long-term treatment, Antibiotic resistance

## Abstract

**Background:**

Chronic obstructive pulmonary disease (COPD) is characterised by progressive development of airflow limitation that is poorly reversible. Because of a poor understanding of COPD pathogenesis, treatment is mostly symptomatic and new therapeutic strategies are limited. There is a direct relationship between the severity of the disease and the intensity of the inflammatory response. Besides smoking, one of the hypotheses for the persistent airway inflammation is the presence of recurrent infections. Macrolide antibiotics have bacteriostatic as well as anti-inflammatory properties in patients with cystic fibrosis and other inflammatory pulmonary diseases. There is consistent evidence that macrolide therapy reduces infectious exacerbations, decreases the requirement for additional antibiotics and improves nutritional measures. Because of these positive effects we hypothesised that maintenance macrolide therapy may also have beneficial effects in patients with COPD who have recurrent exacerbations. The effects on development of bacterial resistance to macrolides due to this long-term treatment are unknown. Until now, studies investigating macrolide therapy in COPD are limited. The objective of this study is to assess whether maintenance treatment with macrolide antibiotics in COPD patients with three or more exacerbations in the previous year decreases the exacerbation rate in the year of treatment and to establish microbial resistance due to the long-term treatment.

**Methods/design:**

The study is set up as a prospective randomised double-blind placebo-controlled single-centre trial. A total of 92 patients with COPD who have had at least three exacerbations of COPD in the previous year will be included. Subjects will be randomised to receive either azithromycin 500 mg three times a week or placebo. Our primary endpoint is the reduction in the number of exacerbations of COPD in the year of treatment.

**Discussion:**

We investigate whether long-term therapy with macrolide antibiotics can prevent exacerbations in patients with COPD. Additionally, our study aims to assess the effect of long-term use of macrolide on the development of antimicrobial resistance and on inflammatory parameters related to COPD. We believe this study will provide more data on the effects of macrolide treatment in patients in COPD and will add more knowledge on its working mechanisms.

**Trial registration:**

http://www.clinicaltrials.gov NCT00985244

## Background

Chronic obstructive pulmonary disease (COPD) is generally accepted to become one of the major health problems in the western worlds in the following years. The main issue is the progressive character of the disease, which is characterised by an ongoing development of non-reversible airflow limitation.

COPD imposes a substantial burden on health-care systems worldwide, as the disease is a major cause of morbidity, mortality, reduced health status and a common cause of medical hospital admission [1]. Because of a poor understanding of COPD pathogenesis, treatment is mostly symptomatic and new therapeutic strategies are limited. One of the known causes of COPD is long-term exposure to noxious particles or gasses. Particularly cigarette smoking is one of the main causes of development of COPD [2]. All smokers show evidence of lung inflammation, but smoking-induced lung injury is variable and appears to be amplified only in a minority of long-term tobacco smokers, suggesting that superimposed processes are the final determinants of COPD development [3,4]. There is a direct relationship between the severity of the disease and the intensity of the inflammatory response [3,4]. Thus, excessive inflammation is likely the key to susceptibility. Inflammation persists long after patients have stopped smoking. The cause of this persistent airway inflammation is unknown although recurrent airway infections seem to play a role in this process.

Macrolide antibiotics have bacteriostatic as well as anti-inflammatory properties [5-7]. The anti-inflammatory capacities of macrolides were firstly established in pulmonary diseases as diffuse panbronchiolitis, a progressive inflammatory disorder of the airways found almost exclusively in Japan [7]. The introduction of long-term macrolide therapy has resulted in dramatic improvements in survival, with 5-year survival rates increasing from 63 to 92% [7,8]. Significant symptom reduction and improved pulmonary function have also been achieved [9-12]. The mechanism of action is thought to be due to immune modifying effects rather than direct antimicrobial activity. Clinical improvement has been reported independent of the presence or absence of chronic airway infection [9] and with antibiotic levels below the minimum inhibitory concentrations of several pathogenic bacteria [13].

Also in patients with cystic fibrosis who are colonised with *Pseudomonas aeruginosa*, macrolide therapy had lead to improvement in forced expiratory volume in 1 s (FEV_1_) and forced vital capacity (FVC), a reduction in exacerbation rate, a reduction in hospital days and days of intravenous antibiotic use, delaying time until the first exacerbation and reducing number of additional courses of antibiotics [14-19]. There is consistent evidence that macrolide therapy reduces infective exacerbations, decreases the requirement for additional antibiotics and improves nutritional measures in patients with cystic fibrosis. A Cochrane review of macrolide therapy concluded that treatment with azithromycin had a small but significant effect on pulmonary function in patients with cystic fibrosis [20].

Although currently the use of maintenance antibiotic treatment in COPD, other than for treating infectious exacerbations COPD, is not recommended by the GOLD report [21]. Several studies have been conducted to assess the effect of long-term therapy with macrolide antibiotics in patients with COPD [22-24]. The results of these studies are conflicting; however some suggest that macrolide antibiotics may become a valuable therapeutic option for COPD patients in preventing exacerbations.

There are essential differences between the different macrolides . From previous studies on macrolide antibiotics it seems that azithromycin is the most potent drug pharmacodynamically [25]. In contrast with erythromycin and clarithromycin, it has a better intracellular uptake, a slower release from cells and a more extensive tissue distribution for a longer lasting effect after cessation of therapy [25,26]. Azithromycin also is known for its mild side effects in comparison with erythromycin [26-29]. Apart from that, in contrast to former studies with macrolides in COPD [22-24] azithromycin can be given on a three times weekly base.

In this randomised placebo controlled trial our main aim is to assess whether maintenance treatment with three times weekly azithromycin in COPD patients with three or more exacerbations in the previous year can decrease the exacerbation rate in the year of treatment and to study the effect of this treatment on microbial resistance.

## Methods

The study is designed as a prospective randomised double-blind placebo-controlled single-centre trial in the department of respiratory medicine in the Amphia Hospital Breda, The Netherlands. Length of study is determined at a period of 3 years, of which 2 years will be spent on patient inclusion and 1 year on treatment. The end of the study is defined by the last visit of the last included subject.

### Patient selection

All patients with COPD who have had three or more exacerbations in the previous year will be asked to participate in the study.

An exacerbation of COPD is defined by a (sub)acute increase of pulmonary symptoms like dyspnoea, coughing, increased sputum volume with or without purulence, for which the patient has consulted a general practitioner (GP) or a respiratory physician, or for which the patient has been admitted to the hospital. The health care professional has judged the symptoms to be in such a degree that treatment was given with systemic steroids and/or a course of antibiotics.

The patients will be recruited from the outpatient department. To assess whether the patient fulfils the criteria for study participation the study subjects’ GP will be contacted to review the patient chart and medication use. The hospital charts will be reviewed as well by the investigator.

### Inclusion criteria

Diagnosis of COPD according to GOLD criteria (FEV1/FVC < 70%), classification into GOLD I (FEV1 80-100% predicted), GOLD II (FEV1 50-80% predicted), GOLD III (FEV1 30-50% predicted) or GOLD IV (FEV1 ≤ 30% predicted).

Age ≥ 18 years.

Three or more exacerbations of COPD in the preceding year of inclusion for which a course of systemic steroids and/or antibiotics therapy was started.

Clinically stable during 1 month. Patients have to be free of COPD exacerbation or respiratory tract infection within a month prior to involvement in the study and in this period they should not have received antibiotics or a course of high doses of systemic steroids defined as more than 10 mg of prednisone a day.

Informed consent.

### Exclusion criteria

Use of antibiotics or a course of high doses of systemic steroids defined as more than 10 mg of prednisone a day within a month prior to involvement in the study.

Addition of inhalation steroids to the patient’s therapy regimen within 1 year prior to study inclusion. Adding inhalation steroids 1 year before trial inclusion can influence the outcome of exacerbation frequencies.

Pregnant or lactating women.

Allergy to macrolides.

Liver disease (alanine transaminase and/or aspartate transaminase levels two or more times the upper limit of normal).

Asthma, defined as episodic symptoms of airflow obstruction which is reversible with bronchodilators, assessed with lung function testing.

Bronchiectasis. A CT scan (1 mm coupes) was performed in all patients to exclude bronchiectasis. Criteria of the BTS guideline Bronchiectasis (non-CF) are used for radiologic definition of bronchiectasis [30].

Malignancy of any kind for which the subject is under treatment or is being monitored as part of follow up after treatment.

Heart failure. A patient is excluded when having clinical signs of heart failure and a cardiac function defined as a left ventricular ejection fraction of less than 45% confirmed by echocardiography or single photon emission computed tomography (SPECT) scan.

Use of drugs that can adversely interact with macrolides and for which therapeutic monitoring cannot be undertaken, e.g. ergotamine derivatives.

### Intervention

Subjects will be randomised to receive either azithromycin 500 mg three times a week or placebo during a 1-year period. During this year subjects will be followed at the outpatient department at 3, 6, 9 and 12 months after initiating the study. During these visits the following tests will be performed according to the flowchart (Table 1):

Lung function testing

Sputum sample collection

Peripheral blood collection

Throat swab

Rectal swab

DS14 questionnaire for assessment of type D personality (only on day 1 and month 12)

Hospital Anxiety Depression Scale (HADS)

12-Item Short Form Health Survey (SF-12)

St. George’s Respiratory Questionnaire (SGRQ).

**Table 1:** Overview of outpatient department visits and tests

	**Day 1**	**Month 3**	**Month 6**	**Month 9**	**Month 12**	Other*
Informed consent	X					
Blood works	X	X	X	X	X	X
Microbiology	X	X	X	X	X	X
Lung function testing	X	X	X	X	X	
Rectal swab	X		X		X	
Questionnaires	X	X	X	X	X	X

In case of exacerbation subjects have the choice to get treatment from their general practitioner or to visit the hospital to be seen by the investigator. Either way sputum and peripheral blood will be collected for immunological and microbiological investigations. Also the subjects with an exacerbation will be asked to complete the SF-12, HADS and SGRQ.

### Type D personality

The Type D Scale (DS14) will be administered to assess Type D personality [31]. This 14-item questionnaire comprises two subscales, Negative Affectivity and Social Inhibition, each consisting of seven items. Items are answered on a 5-point Likert scale, ranging from 0 (false) to 4 (true). A standardised cutoff score ≥ 10 on both subscales is used to classify individuals with a Type D personality [32]. A previous study confirmed that it is the interaction of both traits, rather than the single traits, that incurs an increased risk of adverse health outcomes [32]. Both of the DS14 subscales of Negative Affectivity and Social Inhibition have good internal validity (Cronbach’s *α* = 0.88/0.86), are stable over a 3-month period (*r* = 0.82/0.72), and are independent of mood and health status [31].

### Depressive and anxious symptomatology

The Dutch version of the Hospital Anxiety and Depression Scale (HADS) will be used to assess depressive and anxious symptomatology [33,34]. Both subscales consist of seven items that are answered on a 4-point Likert Scale, ranging from 0 to 3. A cutoff score of ≥ 8 for each subscale represents probable clinical levels of anxiety and depression [35]. Test-retest reliabilities over a 3-week period for the subscales and the total scale are good (0.86 < *r* < 0.91) [34]. The dimensional structure and reliability of the HADS has been shown to be stable across medical settings and age groups [24,34].

### Health status

The Dutch version of the Short-Form Health Survey12 (SF-12) will be administered to assess generic health status [36,37]. This generic instrument measures overall physical and mental health status, as indicated by the Physical Component Scale Summary (PCS) and the Mental Component Summary (MCS) scores [38]. According to standard scoring procedures, all scale scores will be standardised to the general US population (range 0–100, mean = 50, SD = 10), with higher scores indicating better functioning. The SF-12 has been demonstrated to be a reliable and valid instrument [37].

### Health-related quality of life

Disease specific health related quality of life will be measured by the total score on St. George’s Respiratory Questionnaire (SGRQ) [39]. Three component scores are calculated: symptoms, activity, and impacts (on daily life), and a total score. Total scores range from 0 to 100, with lower scores indicating improvement.

### Study endpoints

#### Primary study outcome

Reduction in the number of exacerbations of COPD in the year of treatment.

### Secondary study outcome

Measurement of lung function parameters and 6 min walking distance.

Assessment of presence of type D personality by DS14 questionnaire.

Disease specific health related quality of life measured by St. George’s Respiratory Questionnaire (SGRQ).

Generic health status measured by the 12-Item Short Form Health Survey (SF-12).

Indication of anxiety and depression by Hospital Anxiety Depression Scale (HADS).

Microbiology: Sputum specimens will be cultured. Polymerase chain reaction (PCR) in sputum and serology in serum for viral and atypical microorganisms will be performed. The rectal swabs will be tested for change in rectal flora as a result of maintenance azithromycin.

Measurement of inflammatory markers in serum [erythrocyte sedimentation rate (ESR), C-reactive protein (CRP), pro-adrenomedullin, (pro-ADM), interleukin-6 and cytokine profiles of T-helper 1, T-helper 2 and T-helper 17 cells].

Decrease in percentage of clinical versus outpatient department exacerbations.

Difference in treatment effect between subjects with and without steroid maintenance therapy as hypothesis-generating secondary analysis.

Adverse events of treatment. Symptoms that are believed to be (possibly) related to therapy will be reviewed at the outpatient department. If a patient has an adverse event that is thought to be drug related and that does not resolve, then the patient will be withdrawn from the study. There will be no routine ECG screening since azithromycin (in contrast to erythromycin) is much less likely to be a cause of a prolonged QTc interval.

Length of hospital stay.

Time till first exacerbation.

### Sample size and statistical analysis

#### Power calculation and number of study subjects

This calculation starts with the assumption that the number of exacerbations follows a pure Poisson distribution with a mean rate of 3 per subject per year in the placebo group. A 50% reduction in this rate is considered to be clinically relevant. Hence, in the active treatment group the mean exacerbation rate is set at 1.5 per subject per year. With 33 subjects per treatment group followed up for 1 year, this reduction is detectable with 90% power, given a test size alpha of 0.05 (2-sided).

However, the assumption of a pure Poisson distribution may be too strong. In fact, it is plausible that in this case a zero-inflated Poisson process may be present (the outcome of zero exacerbations has a higher probability than that following from a pure Poisson distribution). In addition there is a risk of overdispersion (the variance is larger than the mean). These phenomena may be expected to have a negative (not exactly quantifiable though) effect on the power of the study.

In order to reasonably compensate for the risk of a too low power and for subjects dropping out within one year follow-up, the number of subjects will be augmented by 40%, so that the sample size is set at 46 subjects per treatment group.

### Statistical analysis

The exacerbation rate (primary efficacy outcome) will be analysed using Poisson regression, with a log link function and the log of the time-under-treatment as offset. The exacerbation rate ratio of active relatively to placebo treatment will be the efficacy parameter of interest that will be tested for significance at the 5% level (2-sided). In addition a 95% confidence interval of this parameter will be calculated. The following baseline covariates will be entered along with treatment group: steroid maintenance therapy, the number of exacerbations in the year preceding randomisation, age, sex, smoking, and the GOLD criteria (Tiffeneau index and FEV1% of predicted). In order to generate hypotheses concerning the modification of the treatment effect by the steroid therapy, the treatment-by-steroid interaction term will be added to the model and its effect will be explored. When necessary, the scale parameter will be used to correct the SEs for overdispersion. Additionally, a zero-inflated Poisson distribution will be fitted to the data in order to test if a better fit is obtained.

Other continuous (secondary) outcome variables with measurements at baseline, 3, 6, 9 and 12 months will be analysed using mixed model ANOVA. The following covariates will be entered in the model along with treatment group: the baseline measurement of the outcome variable, age, sex, smoking and the GOLD criteria. When appropriate, the outcome variables will be suitably transformed in order to obtain normally distributed residuals.

Time to first exacerbation will be analysed using Cox proportional hazards regression with the following covariates entered in the model along with treatment group: age, sex, smoking and the GOLD criteria. Also a Kaplan-Meier curve for time to first exacerbation per treatment group will be presented for illustrative purposes.

Concerning safety, the number and type of adverse event will be compared between the two treatment groups using the chi square (or Fisher’s exact) test.

### Randomisation

All eligible subjects will be randomised using block randomisation sequences generated by computer. Treatment allocation numbers will be entered into individually sealed opaque envelopes. The envelope contains a number that is concealed to the treatment allocation. The allocation list will be kept in a safe in the hospital pharmacy and access is possible by a non-investigator independently. In the event of an emergency medical situation the individual’s randomisation code and group allocation could be identified.

### Ethical aspects

The study has been approved by the ethics committee Toetsingscommissie Wetenschappelijk Onderzoek Rotterdam (TWOR), the ethics committee of the Amphia Hospital Breda and the Centrale Commissie Mensgebonden Onderzoek (CCMO).

The research will be explained in detail (verbally and in writing) to the patient prior to enrolment in the study. The explanation will include the type and method of the research, the tests to be performed, and any potential hazards. An informed consent in writing will be obtained from each patient. The patient can withdraw from the study at any time, without any repercussion for the ongoing care.

The study will be conducted according to the International Conference for Harmonization (ICH) principles of Good Clinical Practice (GCP) and the Declaration of Tokyo (2004). The investigator will conduct all aspects of this study in accordance with all national and regional laws of the pertinent regulatory authorities.

## Discussion

Despite the clinical efficacy of long-term macrolide treatment in a number of respiratory diseases, until recently, only smaller studies had reported on this subject in patients with COPD. These studies showed conflicting results. One study did examine the effect of clarithromycin treatment in a prospective double-blind randomised controlled trial including 67 patients with moderately severe COPD [22]. The effects of 3 months’ clarithromycin therapy on health status, exacerbation rate and sputum bacterial numbers were measured. No significant benefit was seen except for significant improvements in both the St George’s Respiratory Questionnaire symptom score and 36-item short-form health survey physical function score. Another study investigated the influence of erythromycin treatment on exacerbation of COPD [24]. The total number of patients needed for inclusion was not reached; therefore the study was underpowered. However, the data suggested a beneficial effect in number of exacerbations. Recently Albert et al. [23] showed in a large randomised controlled trial that adding daily 250 mg azithromycin to standard therapy reduced the number of exacerbations in patients with COPD. The major concern with this study, raised in a number of comments and editorials, was the question about the development of antimicrobial resistance, which had almost doubled in that trial [40]. Also the question about the working mechanism of azithromycin, whether it has an antimicrobial or immunomodulatory effect, was not answered in that trial.

With the current study we investigate whether long-term therapy with macrolide antibiotics can prevent exacerbations in patients with an instable COPD. Additionally, our study aims to assess the effect of long-term use of macrolides on the development of antimicrobial resistance and on inflammatory parameters related to COPD. We believe this study will provide more data on the effects of macrolide treatment in patients in COPD and will add more knowledge on its working mechanisms.

## Trial status

The trial is currently recruiting study subjects.

## Abbreviations

COLUMBUS: influence of macrolides on exacerbation frequency in patients; COPD: chronic obstructive pulmonary disease; GOLD: The Global initiative for chronic Obstructive Lung Disease; FEV1: forced expiratory volume in 1 s; FVC: forced vital capacity; HADS: Hospital Anxiety Depression Scale; SF-12: 12-Item Short Form Health Survey; SGRQ: St. George’s Respiratory Questionnaire; PCR: polymerase chain reaction; TWOR: Toetsingscommissie Wetenschappelijk Onderzoek Rotterdam; CCMO: Centrale Commissie Mensgebonden Onderzoek; ICH: International Conference for Harmonization; GCP: good clinical practice.

## Competing interests

The authors declare that they have no competing interests.

## Authors’ contributions

SU has contributed to the study protocol and is coordinating, including and monitoring the study participants. RD has participated in the design of the study, contributed to the study protocol and is including study participants. JAJWK has contributed to the study protocol and the design of the study. He is also responsible for the tests required in microbiological section of the trial. NEV has contributed to the study protocol and is responsible for the trial medication. AAEM has participated and coordinated in setting up the laboratory tests. AJP has contributed to the study protocol and the selection of a part of the questionnaires. PM has contributed to the study protocol and is responsible for the statistical analysis. ME has participated in the design of the study and contributed to the study protocol. JGJVA has participated in the design of the study, contributed to the study protocol and is including study participants. All authors read and approved the final manuscript.
